# Obstetrics and Gynæcology

**Published:** 1912-09-07

**Authors:** 


### OBSTETRICS AND GYNÆCOLOGY.

### The Education and Training of the Specialist.

At the present time the two cognate, but essentially distinct, specialties of gynæcology and obstetrics have not very long emerged from a stage of transition which has changed them and the status of those who pursue them almost out of recognition.

We may, first of all, state that it is only within the last two or three centuries that midwifery and gynæcology have been considered proper subjects for men to engage in at all. The "jury of matrons" is now nothing but a picturesque anachronism, which those with any love of their country's ancient history and monuments would not willingly see abolished. But it serves to bring home the fact that the diagnosis of pregnancy, obviously in all ages a problem both of obstetrics and gynæcology, was not entrusted to medical practitioners in mediæval England. In France the same held good; in the seventeenth century one of the Bourbons was fiercely criticised and lampooned for surreptitiously introducing a male accoucheur into the lying-in room of one of his mistresses. Even in comparatively modern days traces of these same customs remain; thus it is stated that at the accouchement of the Duchess of Kent (when Queen Victoria was born, in 1819 that is to say) a midwife alone was present.

It will thus be seen that obstetrics is a fairly recent science (and the same is true of gynæcology). Britain, no doubt, produced eminent obstetricians in the eighteenth century; but it was not until Semmelweiss, O. W. Holmes, and, later, Lord Lister had lived and laboured that the foundations of modern obstetrics as we now know it were laid. Notwithstanding Listerian principles, the old distinction still survives by which an obstetric practitioner is termed a physician and addressed as "Doctor." This in itself is a matter of no particular moment; but the application of the same cast-iron rule to the sister science of gynæcology has been fertile in stumbling-blocks and heart-burnings.

Gynæcology, up to the Listerian epoch, was even more clearly within the province of a physician, as opposed to a surgeon, than was obstetrics. For obstetrics did even in Smellie's day involve a quite considerable amount of operative and manual dexterity; and Barnes's classic on "Obstetrical Operations" is still quoted from occasionally nowadays. But gynæcology was for the most part a matter of pills, potions, and poultices. What little operative gynæcology there was to be done was entrusted as a rule to a general surgeon; the physician-gynæcologist passed his cases on when he judged intervention to be requisite, just as the general physician does to-day. There was, before say 1880, so little operative surgery done in the female pelvis that its development into a separate specialty was not contemplated. Lawson Tait did, it is true, operate on the pelvis through the abdomen before Listerian doctrines were fully appreciated; but he was a brilliant exception.

Then came the rise of surgery, consequent on the revelations of Lister; and operative gynæcology was bound to rise with it. The older men practising as obstetrical and gynæcological physicians were at first distrustful of the innovation, and made no serious attempts to dispute with the general surgeon's abdomino-pelvic surgery. Like Barnes and Matthews Duncan, the two leading gynæcologists in London before 1880, they were content to do occasional vaginal operations and to refer other operative treatment to the surgeons. But vaginal operations could be done with far greater success and safety than before, and also many diseases could be subjected to operative treatment, and cured thereby, which formerly had received only palliative treatment with drugs. Not unnaturally the gynæcological physicians, already entrusted with vaginal operating, began to thirst for greater opportunities. Ancient customs and vested interests for a long time combined to thwart them. But gradually at one hospital after another they were allowed to undertake the entire medical and surgical care of the cases admitted to their wards.

At some institutions the change was stoutly resisted by the general surgeons; indeed, at one or two well-known centres it has not even yet been conceded, we believe. This reactionary policy produced sometimes results which were deplorable, if not unnatural. For the gynæcologists, thus restricted to vaginal routes, devised ingenious methods of removing through that canal offending structures which would have been much better dealt with through abdominal incisions.

### Degrees and Diplomas.

It will thus be understood that the older school of gynæcologists regarded themselves as essentially physicians, whereas the young men of the present day quite rightly demand to be classed as surgeons. As a general rule, those who aspire to a post at a large teaching hospital as physician are expected to provide themselves with an M.D. degree from some reputable University, and with the Membership of the Royal College of Physicians, whereas those who desire election as surgeons refrain from taking an M.D. and become Fellows of the Royal College of Surgeons. Originally, the gynæcological physician was invariably a titular doctor and a member of the College of Physicians. Nowadays as often as not he is F.R.C.S. alone, or sometimes he sits on the fence by obtaining the right to subscribe himself M.D., M.R.C P., F.R.C.S. At some of the hospitals where women alone are treated, the old-fashioned distinctions survive in theory, though not in practice. Thus at one justly famous London hospital there are separate staffs of physicians (with assistant physicians) and surgeons (with assistant surgeons); yet all have similar privileges and work.

It will thus be seen that there is no hard-and-fast rule respecting the qualifications which will be demanded of candidates for hospital appointments as gynæcologists. But the trend of current tendencies is to require the possession of the F.R.C.S. diploma. If this represents finality is doubtful; when the State takes over—as it must some day—the admission of men to the Medical Register, we may expect to see developed some system of qualification and registration of proficiency in special subjects. The ophthalmologists have already attempted something of this kind, though not with any striking success. The diploma in ophthalmology (D.O.) which is granted by Oxford University is an earnest of future developments which must eventually extend to all the recognised specialties. Thus neither the M.R.C.P. nor the F.R.C.S. can be said to constitute any guarantee whatsoever of proficiency in either obstetrics or gynæcology. Both of them, and especially the latter, are severe tests of the candidate's ability to digest encyclopædic text-books.

The real essence of professional efficiency lies in work, knowledge, and experience. The young man newly qualified who is desirous of making a name for himself will be well advised to seek first a house surgeoncy at the best centre of training available; generally this will be that connected with his own school. After filling this post he will certainly profit if he can become house physician, though this is not essential. Before going further he should attain whatever advanced diploma he has in contemplation, generally the Fellowship of the Royal College of Surgeons. He may then obtain a post as resident medical officer at a lying-in hospital if he can; sometimes he will find it advisable to become a clinical assistant there for a space first of all. Following this he should become house surgeon to a first-rate women's hospital. In some of the largest schools it is possible to obtain posts which carry with them both obstetric and gynæcological training at the same time. This course of post-graduate work will take up two or three years. When it is complete, the next thing is to obtain some non-resident appointment, such as that of registrar to a gynæcological department or hospital, or registrar to a lying-in hospital, or obstetric tutor to a medical school. These posts bring further opportunities of study, such as the taking of out-patients and of occasional operative work for members of the staff. In London, at any rate, the competition is not so keen as might be expected. Thus one young gynæcologist at the present moment holds three registrarships and a tutorship, most of them obtained without very serious competition.

When once the aspirant has got to the stage of holding registrarships, his next care must be to ensure his selection for the next vacancy on the staff. As far as professional reputation with his colleagues goes, probably no gift is more valuable than that of fluency with the pen; as far as worldly reputation is concerned, perfect tact and manners are the all-important attributes.

